# Assessing resident experience of a new experiential learning health advocacy curriculum: a mixed methods study

**DOI:** 10.1186/s12909-024-05961-7

**Published:** 2024-09-11

**Authors:** Aliza Moledina, Sydney Ruller, Samantha Halman, Yvonne Ying

**Affiliations:** 1 Department of Medicine, University of Ottawa, 501 Smyth Road, K1H8L6 Ottawa, ON Canada; 2 Division of General Internal Medicine, The Ottawa Hospital, Ottawa, Canada; 3 Clinical Epidemiology Program, Ottawa Hospital Research Institute, Ottawa, Canada; 4 Department of Surgery, University of Ottawa, Ottawa, Canada; 5 Department of Surgery, Children’s Hospital of Eastern Ontario, Ottawa, Canada

**Keywords:** Health Advocacy, Health equity, Experiential learning, Medical Education

## Abstract

**Background:**

Health Advocacy is considered one of the most difficult skills to teach. Many medical learners feel ill-equipped in social competencies and identify it as a significant gap in their medical training. Experiential learning has recently been emerging as a more effective method to teach health advocacy. The Post Graduate Medical Education (PGME) Health Advocacy Day is a new experiential learning curriculum designed to teach important competencies of health advocacy and social accountability to post-graduate medical residents at the University of Ottawa in Ottawa, Canada. The objective of this mixed-methods study was to assess resident experiences.

**Methods:**

Second-year trainees from all adult residency programs attended the Advocacy Day as part of a mandatory academic day. All participants completed a mandatory pre-and post-session quiz to assess knowledge of key topics before and after the course. We also distributed a voluntary survey to all participants and invited residents to participate in semi-structured interviews to provide feedback on the course. We used descriptive statistics to analyze quiz scores and survey results and conducted a paired t-test of pre and post-test quiz scores. We also performed a thematic analysis of qualitative feedback, specifically survey comments and semi-structured interviews.

**Results:**

One hundred and eighty-three residents participated in the Advocacy Day and 112 (61.2%) completed the post-course survey. Ten residents volunteered to be interviewed. Respondents were generally satisfied by the session and felt it was of good quality. Most residents felt the course enhanced their ability to advocate for individual patients or communities (*N* = 80; 71.5%) and understand patients and families’ lived experience with illness (*N* = 87; 77.5%). Most residents also felt the course improved their knowledge of the impact of social determinants of health (*N* = 91; 81.2%) and increased their awareness of local resources that can support patients and their families (*N* = 88; 78.3%). Visiting community sites in-person and meeting persons with lived experiences were highlighted as the most valuable components of the course.

**Conclusion:**

Experiential learning can be integrated within post-graduate medical curricula to teach health advocacy competencies. Future studies should examine the longitudinal impact of the curricula, to determine whether shifts in perspectives persist over time.

**Supplementary Information:**

The online version contains supplementary material available at 10.1186/s12909-024-05961-7.

## Background

Health advocacy is a core competency in the CanMEDS framework, a model that outlines the essential roles and skills that physicians require, to meet the healthcare needs of the community [1, 2]. However, despite broad recognition of the importance of social determinants of health, health advocacy training is still under-represented in medical education [3–7]. As a result, many learners feel ill-equipped in social competencies, identifying them as a significant gap in their medical training [4, 7–9]. 

Experiential learning has emerged as an effective method to teach health advocacy [10–15].“Hands-on” learning and community engagement allow learners to observe health disparities firsthand, and better appreciate the challenges that members of the community face in accessing care [11, 12, 16, 17]. Community-service learning (CSL) projects can play a positive role in increasing compassion and respect for the underserved, enhancing cultural understanding and improving communication with diverse patients [11, 13, 18] Notably however, though trainees experience a significant drop in empathy once they enter the clinical setting, post-graduate advocacy training is still widely variable, and often lacks curricular focus [14, 19–22]. Given insufficient formal teaching, trainees often feel that the onus is on them to learn core health advocacy competencies, leading to the perception that advocacy is not as important as other skills [20].

In response to these gaps in training, the University of Ottawa in Ontario, Canada, implemented a new faculty-wide experiential learning curriculum to improve health advocacy training for postgraduate residents. The curriculum, entitled Health Advocacy Day, involves 3 key components: (1) visiting community sites and engaging with front-line-service providers to learn about local programs and resources (2) meeting persons with lived experience and role play to better understand the barriers specific patients face and (3) knowledge consolidation with group presentations and pre-and post-course quizzes. The objective of this mixed-methods study was to assess resident experiences of the new curriculum.

## Methods

### The intervention (course description)

The course was inspired by a similar initiative run by the Northern Ontario School of Medicine Paediatrics program, as well as a subsequent initiative run by University of Ottawa Paediatrics program. Over 30 staff, faculty and trainees from the University of Ottawa and affiliated hospitals and health centers with expertise in health advocacy participated in course development, with input from community partners. A dry run of all scenarios was conducted between July and August 2021, to ensure all modules were logistically sound.

We ran the Advocacy Day five times during the 2021–2022 academic year. Second-year trainees from all residency programs except Paediatrics, attended the course as part of a mandatory academic day. Clinical fellows were also offered course spots when available. Paediatric residents were excluded as their program had already implemented a similar curriculum focused on child health services.

We placed residents in groups of 4–6 and randomly assigned a topic to each group one week before the course. We also e-mailed residents reflective questions and a pre-course quiz.

In the morning session, residents participated in learning activities to better understand the barriers patients face. For example, residents assigned to the poverty group were given a limited budget to purchase a week of groceries for an entire family. Residents also visited a community organization to learn about local services that support these individuals. At most sites, they also met with one or more persons with lived experience. Group topics and community organizations visited is listed in Table 1.

**Table 1:** PGME Health Advocacy Day group topics and organizations visited

Topic	Examples of Community Organizations visited
Alcohol Use Disorder	- Residential managed alcohol program - Centre for emergency detoxification
Chronic Illness	- Chronic illness and Healthy Living community support group - Hospital dialysis program
Elder care/Dementia	- Adult day program and respite care - Long-term care residence
Homelessness	- Medical organization serving inner city residents - Shelter for persons experiencing homelessness
Indigenous Health	- Primary care clinic focused on Indigenous patients - Regional Indigenous cancer care program
LGBTQ2S+	- Organization that supports trans youth
New Immigrants	- Charity that helps support new immigrants
Opioid Use Disorder	- Safe injection site - Safe supply program
Poverty	- Community food bank
Refugee health	- Refugee resettlement center that provides short term housing for government sponsored refugees upon arrival

* 7 to 9 topics were run each session depending on community partner availability

Though topics were assigned randomly, residents could request to opt-out of specific topics, to avoid areas that could be triggering or in which they already had expertise. Ten residents requested to opt-out of a topic and were thus re-assigned. Residents did not have to provide a reason for their request to opt-out.

In the afternoon, each group delivered a 10-minute presentation to their peers to share information from their morning visit. The presentations allowed residents to learn about all topics rather than only the one they had been assigned. At the end of the day a post-course quiz was given to trainees. Both pre-and post-quizzes included one question per topic covered.

### Overall study design

All residents who attended Advocacy Day were enrolled in the study as part of a quality improvement initiative. A sequential mixed methods evaluation was conducted as follows:

First, we asked residents to complete a mandatory pre-and post-session quiz to assess how knowledge of key topics changed after attending the course. Second, we distributed a voluntary online survey to all participants at the end of the day, consisting of both Likert scale and free text questions. Lastly, we invited residents to participate in a voluntary semi-structured interview. The interviews were between 15 and 40 min in length and explored resident perceptions of the course and its’ role in teaching health advocacy. Residents who volunteered to participate in the interview received a $25 gift certificate for their time.

The survey and interview questions are presented in Appendix A.

This project was exempted from full institutional ethics review given the quality improvement focus.

### Quantitative data analysis

We used descriptive statistics to analyze de-identified quiz scores and survey results. A paired t-test of pre and post-test scores was performed using SAS 9.4.

### Qualitative data analysis

The interviews were professionally transcribed verbatim. Two members of the study team (A.M, S.R.) used iterative methods to code the qualitative data using NVivo 20.7.1. We analyzed both the free-text questions in the survey as well as the semi-structured interviews. We stopped recruitment for interviews once thematic saturation was achieved. Once themes were identified, they were shared with the broader team to reach consensus. We then selected quotes that were felt to best represent the narratives that emerged from the interviews. All audio and video data were de-identified prior to analysis.

## Results

One hundred and eighty-three residents participated in the Advocacy Day. One hundred and eighty residents were in their second year of post-graduate training and 3 were more senior residents.

Of the residents who participated, 112 (61.2%) completed the post-course feedback survey and 10 (5.5%) volunteered to be interviewed (Table 2).

**Table 2:** Self-reported specialties of residents who participated in the PGME Health Advocacy Day survey or interview

	Survey (total *N* = 112) *n* (%)	Interviews (total *N* = 10) *n* (%)
Surgical Specialty	13 (11.6%)	2 (20.0%)
Medical Specialty (Family Medicine, Internal Medicine, Neurology)	70 (62.5%)	4 (40.0%)
Diagnostic Specialty (Lab medicine, Pathology, Radiology)	9 (8.1%)	1 (10.0%)
Acute Care (Anesthesia, Emergency Medicine)	10 (8.9%)	0 (0.0%)
Other (Psychiatry, Physical Medicine and Rehabilitation, Radiation Oncology, etc.)	10 (8.9%)	2 (20.0%)

### Overall quality of the session

Respondents generally felt satisfied by the session and felt that it was of good quality with a good level of interactivity (Table 3). The most common concern was limited time to prepare the afternoon presentation. The participants who gave the session lower quality ratings noted that meeting the community partners in-person and discussing with patients to be the most effective parts of the day. Of the 12 participants who rated the session as only “fair”, 7 still felt that the session would help them advocate more effectively in the future.

**Table 3:** Overall impressions of PGME Health Advocacy Day, as reported by University of Ottawa residents who completed the post-session feedback survey (*n* = 112)

Question *N* = 112	Not at all important	Slightly Important	Moderately Important	Very Important	Extremely Important
How relevant do you think health advocacy is to the future practice of health care providers?	0.9%	0.9%	9.8%	33.9%	54.5%
How important is it that health care providers receive training regarding the root causes of social disparities and their impacts on health?	0.0%	1.8%	10.8%	28.8%	58.6%
	**Very Poor**	**Poor**	**Fair**	**Good**	**Excellent**
How would you rate the overall quality of today’s session?	0.0%	0.0%	15.2%	49.1%	35.7%
How would you rate the format of the session?	0.0%	2.7%	18.8%	57.1%	21.4%
How would you rate the interactivity of the session?	0.0%	1.8%	15.2%	49.1%	33.9%
	**Not at all Satisfied**	**Slightly Satisfied**	**Moderately Satisfied**	**Very Satisfied**	**Completely Satisfied**
To what extent did this session meet your learning needs?	0.9%	7.1%	29.5%	44.6%	17.9%

Four major themes were identified from the semi-structured interviews and free-text comments: the importance of health advocacy, the most effective elements of Advocacy Day, areas of debatable effectiveness, and reflections on clinical applicability and lessons learned.

### The importance of health advocacy

The majority of course participants felt that health advocacy was very to extremely important to the future practice of health care providers (88.4%) and that it was very important or extremely important that health care providers receive dedicated training on the root causes of social disparities and their impact on health (87.4%) (Table 3). All individuals who participated in the interviews (*n* = 10) felt health advocacy was very important within medicine.

### Prior advocacy training

In general, residents interviewed felt there was little formal teaching around health advocacy during residency, and that formal advocacy training was more common in medical school. Most interviewees described advocacy training in residency as primarily informal; for example, through caring for marginalized patients in hospital or in outpatient clinics. One resident described this informal training, stating:

*[O]ther physicians I work with who will give you little tips about how to kind of get around a certain limitation when it comes to prescribing certain medications*,* or how to really support your patients*,* saying*,* “Oh*,* this particular organization will provide*,* you know*,* X amount of therapy.”. so it’s kind of learning on the job*,* from that perspective*,* for these little extra tips.* (Interviewee #4)

As with most informal learning however, residents emphasized that this training was largely dependent on the preceptor each resident was assigned to.

### Most effective parts of advocacy day

The value of speaking with patients with lived experience and visiting community sites was by far noted to be the most effective part of the Advocacy Day in both the survey and interviews. One participant highlighted this by saying:

*I think number one*,* the fact that we actually got to interact with the community partners and meet people who are affected by the challenges that we were learning about. Because this is more of an experiential experience where you actually connect more with the problem and with the topic. (*Interviewee #2*)*

Visiting community sites in-person was emphasized as a strength of the program, as it provided a way to better visualize the challenges that patients face. For example, residents who visited the shelter stated it was valuable to physically see the environment that patients lived in:

*“[Most] Valuable - being able to physically see the shelter system to understand what environment we discharge patients from the hospital to. (*Survey ID 110009207056*)*

*“[…]just like stepping into one of the rooms at the shelter. And then*,* you know*,* I hadn’t really realized that you’d be sleeping with six other people in the room in really tight bunkbeds and things like that[…]it’s just a very clear way to exemplify some of the challenges that some people face.* (Interviewee #4)

Multiple respondents highlighted that seeing sites in-person also provided a deeper appreciation for community supports and would allow them to better advocate for use of these resources in the future. One participant described that she now better understands the services offered by a local opioid use disorder program, which would help her motivate her patients to seek help in the future. She stated:

*“I’ve had patients before… who have suffered from opioid use disorder and the question is always …. where to go from there[…] [for example] a week before the session [we had a patient] where we were like oh*,* I think < organization > might have a program that could be good for this person. But I had never really heard of it before*,* so we gave him all the information but I think now knowing everything that they offer and how they have this walk-in format and they’re quite accessible during the week*,* and knowing their hours*,* it’s a lot easier to hopefully motivate the patient to follow up with this clinic*,* knowing everything that they offer.”* (Interviewee #6).

Visiting community sites in-person and meeting persons with lived experience were thus highlighted by residents as the most valuable part of the course.

### Areas of debatable effectiveness

The residents’ most common concern was feeling rushed to create their presentations after being in the community all morning. Others also described time pressures in completing all the morning activities, as sometimes they had multiple destinations to go to. One survey respondent highlighted this concern saying,

:*[…]little time between our morning activity and then having to prepare a presentation over lunch prior to the afternoon was difficult (*Survey ID 110008324543*)*.

The residents also had mixed feelings on the effectiveness of the afternoon peer-delivered presentations. Many participants commented that the presentations were the least effective part of Advocacy Day, citing limited interaction and variability in what was presented by each group. In lieu of presentations, some participants commented that they would have instead preferred either more informal and reflective group discussions or the opportunity to visit an additional community site. One resident stated that the in-person visits were better suited to teaching health advocacy competencies, saying:

*Having an afternoon of presentations and having to prepare a presentation takes away from our learning opportunities to engage with community partners. I think if we had more time to visit more sites throughout the day and avoid spending 3 h sitting through presentations would be better. I really enjoyed my in-person tours - it really helped me understand the realities of patients better*,* along with how to be a better physician to help allied health in the community (*Survey ID 110010673559*)*.

However, some participants did feel the presentations were effective as a learning tool, particularly in consolidating knowledge and conveying practical knowledge on local resources. One resident stated:

*[…]the most useful thing that I got from the presentation is specifically names of resources*,* organizations that exist. Like more practical things. (*Interviewee #6)

Some participants acknowledged that the presentations were a means by which practical information on a large range of topics could be delivered.

There was also mixed commentary on the effectiveness of the role play scenarios. Some respondents felt the experiential learning scenarios were helpful to be able to appreciate challenges patients face. For example, one participant described the difficulty he faced in trying to take a bus with a wheelchair:

*[S]omething simple for me was just taking a bus with a wheelchair*,* […] I was like wow*,* this is eye-opening in a different way. (*Interviewee #3*)*

However, others did not feel there was value in these scenarios. One resident stated that role-playing was not required to empathize with a marginalized patient’s experiences:

*I felt that the scenarios were unnecessary – I do not have to role-play a certain group to be able to empathize or consider their experiences.* (Survey ID 110009207237)

There was overall limited information on the role play elements as only a small number of respondents commented on this explicitly when providing feedback.

### Reflections on clinical applicability and lessons learned

All residents (*n* = 183) who participated in Advocacy Day completed the mandatory pre- and post-session knowledge test. The mean pre-test score was 69.7 (S.D. 17.8) and the mean post-test score (using the first test attempt) was 92.7 (S.D. 9.3). A paired t-test of pre and post-test scores was statistically significant (*p* < 0.0001) indicating significant improvement in knowledge of health advocacy topics after completion of the course.

Of the 112 residents who completed the feedback survey, most felt the course improved their ability to support patients and families with community service navigation (*N* = 76; 67.9%), advocate for individual patients or communities within and beyond the clinical environment (*N* = 80; 71.5%) and understand patients and families’ lived experience with illness (*N* = 87; 77.5%) (Table 4). Similarly, most participants felt that the course improved their knowledge of the impact of the social determinants of health (*N* = 91; 81.2%) and the local community resources available to support patients and their families (*N* = 88; 78.3%) (Table 4).

**Table 4:** Lessons learned from PGME Health Advocacy Day, as reported by University of Ottawa residents who completed the post-session feedback survey (*n* = 112)

Question *N* = 112	1 (Least Helpful)	2	3	4	5 (Most Helpful)
**Has this session improved your confidence and your ability to:**					
Support patients and families with community service navigation	0.0%	5.4%	26.8%	50.0%	17.9%
Respond to an individual patient’s health needs by advocating with the patient within and beyond the clinical environment	0.0%	4.5%	24.1%	53.6%	17.9%
Respond to the needs of the communities or populations they serve by advocating with them for system-level change in a socially accountable manner	0.0%	6.3%	25.0%	54.5%	14.3%
Understand patients’ and families’ lived experiences with illness	0.0%	4.5%	18.0%	46.0%	31.5%
**Has this session improved your knowledge of**					
Patients’ and families’ lived experiences with illness	1.8%	2.7%	19.6%	45.5%	30.4%
Factors that influence disparities in accessing optimal health and health care	1.8%	0.9%	15.2%	50.0%	32.1%
Local community resources available to support patients and families	0.9%	1.8%	18.9%	43.2%	35.1%
The social determinants of health and their impact on achieving optimal health	0.9%	1.8%	16.1%	49.1%	32.1%

In the survey comments and the qualitative interviews, participants described three ways that they will bring what they learned to their clinical practice.

Firstly, they described a greater understanding of the barriers that some patients face in navigating the health system, and a better appreciation of the role of social determinants of health. For example, one participant stated learning about challenges patients face in affording transportation to dialysis was an “eye-opening” experience, where-as another participant shared insight he gained about the difficulties refugees face when they don’t speak the language:

*The illustration of the lack of funding for transport to dialysis was an eye-opening discussion illustrating the strong social component to medical treatment that we don’t encounter on day-to-day rotations […].* (Survey ID 110008517728)

*We talked to a refugee*,* and she was saying[…]it’s hard when you don’t speak the language. You don’t understand the [bus] routes and whatnot [to get to appointments]. [And we] don’t necessarily think of oh*,* they missed their bus or it was hard…* (Interviewee #9).

Speaking with patients and community members directly allowed residents a different perspective on the health system, in appreciating how external factors impact an individual’s ability to access quality care.

Secondly, residents recognized ways that physicians can advocate for patients, both through clinical encounters (i.e. screening for social factors in the clinical history) as well as in connecting marginalized patients with external community resources. For example, one resident described feeling more competent in identifying individuals at high-risk of intimate partner violence, while another recognized the importance of assessing financial status when prescribing certain medications:

*I now better understand the value in screening for intimate partner violence*,* can better identify who may be at higher risk and thus select those to screen*,* and what resources are available and how to get in touch with them. I also feel less uncomfortable with the idea of broaching the topic with patients.* (Survey ID 110008324762)

*I think I would spend more time in getting a social history[…] right now I’m on thrombosis*,* so a lot of the anticoagulants are not covered unless you have private drug insurance. I’ve become more comfortable in even just asking people*,* what they do for a living*,* how do they afford medication? As a way to get more information about a person. As opposed to*,* like*,* “Oh*,* you have a blood clot. You need this drug.” And then kind of just leave. (*Interviewee #1)

In learning more about the barriers patients face, residents were thus able to identify how they may better serve these patients, within the physician-patient relationship.

Lastly, participants highlighted specific skillsets that physicians should demonstrate in treating patients from marginalized groups. For example, in being more patient and understanding, providing culturally competent and trauma-informed care, and being self-aware of their own biases. Some residents described these lessons, saying:

*I think*,* the biggest takeaway that I had from the Health Advocacy Day was actually the importance of trauma informed care[…] Understanding not just why people use opioid but their life story and how did they become who they are*,* through a trauma informed lens. It’s really helpful to break down that barrier between patients and physicians and also just to be compassionate and relatable to them[…]how people don’t just make decisions because*,* like*,* oh*,* you know*,* they don’t know any better or they’re not smart enough. There’s a lot of things that have happened in their life or are happening in their life that caused them to react or choose or decide in a certain way. (*Interviewee #1)

*Cultural competency in how to respectfully interact with patients of special/vulnerable populations e.g. transgender or Inuit patients* (Survey ID 110008880935).

Residents were thus able to recognize the diversity of the patient population, and the distinct challenges that certain members of the community face. In doing so, they recognized the need for health care providers to adapt the provision of care accordingly.

## Discussion

Health advocacy is one of the most difficult CanMEDS roles to teach, and traditional methods of teaching and evaluating competency are not meeting trainees’ needs. [7–9, 23–25] Though community service learning and other experiential activities are increasingly being incorporated at the undergraduate level, these activities cannot easily be extrapolated to the post-graduate setting, due to distinct pressures in clinical responsibilities and time [26]. 

Our unique experiential learning curriculum exposed post graduate medical trainees from a wide range of specialities to core health advocacy topics. Though the course was adapted from paediatrics initiatives, our university wide roll-out demonstrated that experiential curricula can be adapted to serve residents across all specialties. Though organization of the course was labour intensive, having a faculty-wide initiative prevented duplication of effort between individual programs, and promoted the exchange of ideas between residents from different specialties.

Residents who participated in the course highlighted the benefit of meeting persons with lived experience and visiting community sites, in improving their understanding of the impact of social determinants of health as well in appreciating local resources. Residents reported a deeper understanding of how physicians can advocate for patients, with some also acknowledging the need for specific skillsets to effectively treat patients from marginalized groups. Knowledge of key topics also improved, as demonstrated by higher quiz scores after completing the course. The most frequent criticism of the course was the time pressure to prepare the afternoon presentation. Based on the initial feedback we adapted subsequent sessions to allow for more preparation time between activities.

Our course adds to existing literature in demonstrating the benefit of experiential learning in teaching core health advocacy competencies. Experiential learning allows a trainee to gain knowledge and skills that are difficult to attain in the classroom setting and is more likely than didactic learning to lead to changes in behaviours and attitudes. [17] Reflexivity and engagement are crucial to understand the barriers faced by marginalized communities, including meaningful discussions on the impacts of intersectional discrimination and implicit bias. [27] Our curriculum offers an effective and practical approach of teaching health advocacy, whereby a large number of post-graduate trainees can engage with underserved populations and community partners in an experiential but structured format.

There are some limitations of our study. Firstly, the study only involves a single institution. However, the program was attended by residents from all adult specialties which increases confidence in the results. Secondly, given both the survey and interviews were not mandatory, volunteer bias may have impacted the results. It is reassuring however that compared to many online surveys, the response rate of our survey was high (61%) and included many written comments. The similarities in themes between the survey comments and the interviews further support the validity of the results. Lastly, our analysis did not compare the experiences of residents who were assigned to different topics; it is possible that some groups were more effective than others.

Given mixed reviews on the benefit of the afternoon presentations, we recommend that future studies examine whether other methods of sharing and consolidating data from experiential learning experiences may be more effective. Reflective practices are a pillar of experiential learning and should be closely paired with core learning objectives. [17] Unfortunately, language used to describe core objectives within the health advocacy competencies is often vague or not sufficiently measurable [20]. Developing a clear, shared vision of health advocacy, and clarifying the key advocacy competencies that trainees need to acquire should thus be prioritized by post-graduate medical educators, to better guide training and evaluation processes [4, 20, 23]. 

Future studies should examine the long-term impact of the course. Though residents highlighted several ways the session would be clinically applicable, it is important to assess whether shifts in perspective persist over time, particularly as residents return to their challenging and high-stress clinical environments.

## Conclusion

Despite being recognized as a core CanMEDs competency, post-graduate health advocacy training remains highly variable, often dependent on the preceptors that a learner is assigned to. Experiential learning has emerged as an alternate approach for teaching health advocacy competencies. The new Advocacy Day at the University of Ottawa strove to address gaps in training by developing an experiential learning curriculum for post-graduate trainees. The curriculum was designed to enhance understanding of the impacts of social determinants of health and to familiarize trainees with community resources that can support marginalized patients and their families. The program was well-received, with residents emphasizing the value of engaging with individuals with lived experiences and community partners to strengthen their health advocacy skills. Future studies should examine the longitudinal impact of the course, to determine whether shifts in perspectives persist over time.

## Electronic supplementary material

Below is the link to the electronic supplementary material.

Supplementary Material 1

## Data Availability

The datasets used and/or analyzed during the current study are available from the corresponding author on reasonable request.

## Abbreviations

CSL: Community Service learning
LGBTQ2S+: Lesbian, Gay Bisexual, Transgender, Queer or Questioning, Two-Spirit, and additional sexual orientations and gender identities
PGME: Post Graduate Medical Education
