# Supplementary material for: Assessing resident experience of a new experiential learning health advocacy curriculum: a mixed methods study
Source: BMC Med Educ. 2024 Sep 11;24:988. doi: 10.1186/s12909-024-05961-7 (PMC11391829; doi:10.1186/s12909-024-05961-7)
Supplement: Supplementary file 1 — Supplementary Material 1 [file 12909_2024_5961_MOESM1_ESM.docx]

**Appendix A -**Survey and Interview Questions

Survey Questions

1. Please indicate your specialty
   1. Surgical specialty
   2. Medical specialty – Family medicine, internal medicine, neurology etc
   3. Diagnostic specialty – Lab medicine, pathology, radiology etc
   4. Acute care – anesthesia, emergency medicine etc
   5. Others – psychiatry, physical medicine and rehabilitation, radiation oncology etc
   6. Other (please specify)
2. How would you rate the overall quality of today's session?
   1. Very Poor
   2. Poor
   3. Fair
   4. Good
   5. Excellent
3. How would you rate the format of the session?
   1. Very Poor
   2. Poor
   3. Fair
   4. Good
   5. Excellent
4. How would you rate the interactivity of the session?
   1. Very Poor
   2. Poor
   3. Fair
   4. Good
   5. Excellent
5. To what extent did this session meet your learning needs?
   1. Completely Satisfied
   2. Very Satisfied
   3. Moderately Satisfied
   4. Slightly Satisfied
   5. Not at All Satisfied
6. What elements of the session were the most/least effective? In the space provided, please comment on at least one element of the session that you found valuable, and at least one element of the session that you think could be improved.
   1. [Open text]
7. How relevant do you think health advocacy is to the future practice of health care providers?
   1. Not at all important
   2. Slightly important
   3. Moderately important
   4. Very important
   5. Extremely important
8. How important is it that health care providers receive training regarding the root causes of social disparities and their impacts on health?
   1. Not at all important
   2. Slightly important
   3. Moderately important
   4. Very important
   5. Extremely important
9. On a scale of 1-5, with 1 being the least helpful, has this session improved your confidence and your ability to:
   1. Support patients and families with community service navigation
   2. Respond to an individual patient’s health needs by advocating with the patient within and beyond the clinical environment
   3. Respond to the needs of the communities or populations they serve by advocating with them for system-level change in a socially accountable manner
   4. Understand of patients’ and families’ lived experiences with illness
10. On a scale from 1-5, with 1 being the least helpful, has this session improved your knowledge of:
    1. Patients’ and families’ lived experiences with illness
    2. Factors that influence disparities in accessing optimal health and health care
    3. Local community resources available to support patients and families
    4. The social determinants of health and their impact on achieving optimal health
11. What element of today’ session was the most impactful for you?
    1. [open text]
12. Has participation in today’ session impacted on your commitment to integrating health advocacy and social accountability into your teaching, activities or work?
    1. [open text]
13. Will your participation in this session help you to advocate more effectively in the future? Explain why or why not.
    1. [open text]
14. What specific aspects of what you learned today are you most likely to apply in the future?
    1. [open text]
15. Any other comments or suggestions you would like to share?
    1. [open text]

Interview Questions:

1. What does health advocacy mean to you?
2. Do you feel that health advocacy is an important part of being a physician?
3. What health advocacy training have you received thus far, either formally as part of your speciality program, or informally?
4. What did you learn from the PGME Health Advocacy Day? Did the program affect your view of what it means to be a health advocate?
5. What parts of the program did you feel were the most effective in learning how to be a health advocate?
6. Which parts of the program were the least effective in learning how to be a health advocate?
7. If not addressed in responses to Q4/Q5:
   - Did you feel that meeting community partners and/or hearing lived experiences from patients was helpful or beneficial for you? Why or why not?
8. Will what you learned during the PGME Health Advocacy Day affect your current or future practice as a physician? If yes, how so? If not, why not?
   - Can you provide an example of a way you have used (or will use) what you learned during the PGME Health Advocacy Day in your clinical practice?
9. If not answered in responded Q4-8
   - Did you find the presentations component of the day to be beneficial for your learning? Did any of the presentations change your practice or views? How effective were these presentations compared to other health advocacy lectures delivered in medical school or residency?
10. Health Advocacy is an important CanMED role that is often difficult to teach and learn. What further resources or supports would aid you to become a better health advocate?
